# Developmental Toxicity of Micro(Nano)Plastics (MNPs) Exposure in Mammals: A Mini-Review

**DOI:** 10.3390/toxics13030224

**Published:** 2025-03-19

**Authors:** Gongxiang Xia, Teng Wan, Zhuan Chen, Cuiqing Liu, Ran Li

**Affiliations:** School of Public Health, Zhejiang International Science and Technology Cooperation Base of Air Pollution and Health, Zhejiang Chinese Medical University, Hangzhou 310053, China; 202112213603010@zcmu.edu.cn (G.X.); 202211115611008@zcmu.edu.cn (T.W.); 202211115611002@zcmu.edu.cn (Z.C.); liucuiqing@zcmu.edu.cn (C.L.)

**Keywords:** micro(nano)plastics, developmental toxicity, mammals, placental barrier, neurodevelopmental toxicity

## Abstract

Micro(nano)plastics (MNPs) pose a significant threat to both ecological environments and human health. This review systematically examines the developmental toxicity of MNPs in mammals, with a particular focus on the impact of maternal and paternal exposure on offspring. Evidence indicates that MNPs can cross placental barriers, inducing abnormal development of embryos, fetuses, and placentas. This disruption leads to a range of adverse outcomes, including neurodevelopmental abnormalities, behavioral disorders, reproductive system damage, etc., in offspring. Through a comprehensive analysis of the existing literature, this review aims to provide a foundation for future research on the developmental toxicity of MNPs and highlight the urgent need for action to mitigate the detrimental effects of MNPs on human health and ecosystem integrity.

## 1. Introduction

**Figure 1:**
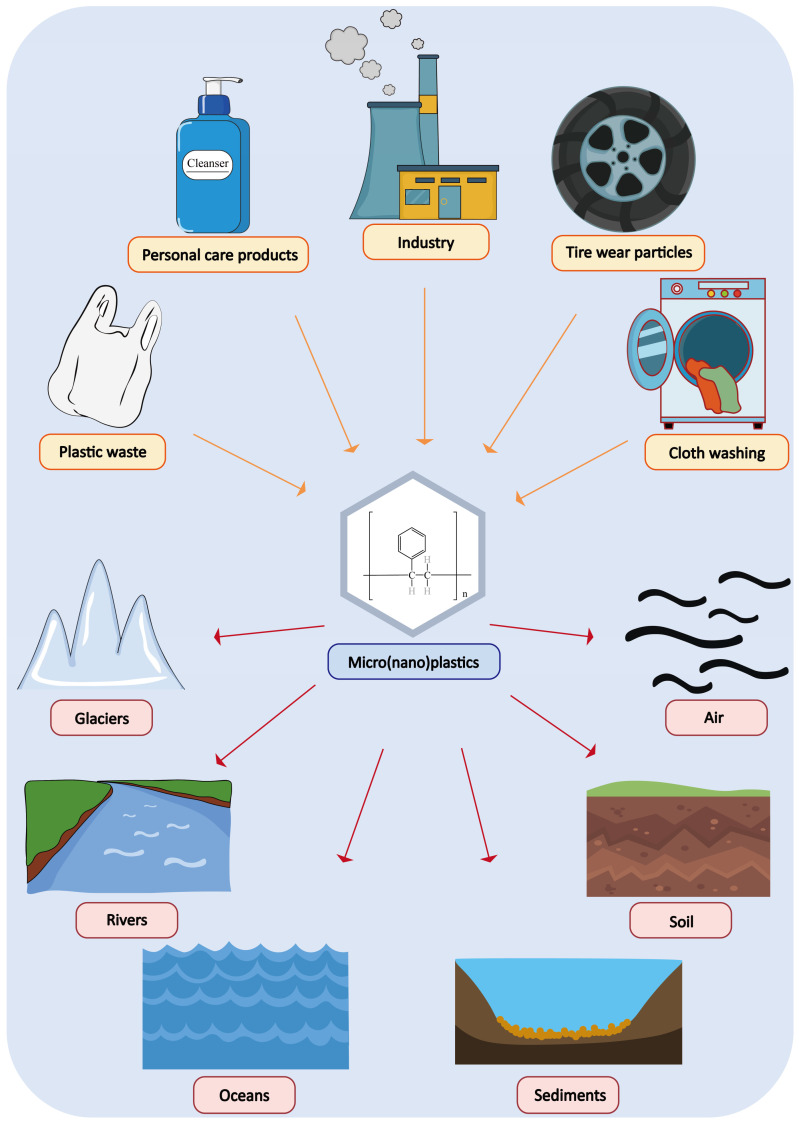
Sources of micro(nano)plastics in the environment and their pathways of distribution.

Plastic products are widely used in various industries, including packaging, construction, textiles, transportation, electrical and industrial machinery, due to their versatility, durability and affordability [1]. Over the past decades, plastic consumption has surged to several hundred million metric tons, with projections indicating a potential increase to 800 million metric tons by 2050 [2]. However, only about 10% of plastic waste is recycled [3]. The extensive use and improper disposal of plastic items have led to the breakdown of plastics into micro(nano)plastics (MNPs), which refer to plastic particles or fibers that range in size from nanometers (≥1 nm) to micrometers (≤5 mm), through physical weathering, ultraviolet (UV) radiation, and biological processes [4,5,6]. MNPs have become almost ubiquitous in the environment [7]. So far, they have been detected globally in marine and terrestrial ecosystems, including oceans, rivers, air, drinking water, sediments, and glaciers [8,9,10] (Figure 1). The widespread presence of MNPs raises significant concerns about their potential threats to human health and other organisms. They can affect biota, disrupt ecosystem functioning, and pose risks to humans through bioaccumulation and biomagnification [11,12,13,14]. Therefore, studying the potential toxicity of MNPs is of great significance.

Developmental toxicity refers to the adverse effects on the growth, differentiation, and survival of cells, tissues, organs, and whole organisms that occur during development [15]. These effects can be characterized by abnormal structure, functional defects, or changes in growth patterns [16]. Given the widespread presence of MNPs in various ecosystems, it is urgent to understand their impact on biological health, particularly during the developmental stages of mammals. MNPs are small in size and can more easily cross biological barriers, which may lead to their systematic distribution and accumulation in target organs. This raises concerns about their capacity to disrupt normal developmental processes, which are critical for the health and survival of individuals and populations.

This review systematically examines the existing literature on the developmental toxicity of MNPs in mammals, focusing on the impact of maternal or paternal exposure to MNPs. It encompassed a range of toxicological effects, including general developmental toxicity, neurodevelopmental toxicity, offspring’s reproductive toxicity, etc. Potential mechanisms of developmental toxicity induced by MNPs include oxidative stress, inflammation, metabolic disruption, and apoptosis. Through elucidating the developmental toxicity of MNPs, this review aims to provide a comprehensive overview of the current state of knowledge, identify key knowledge gaps, and highlight the need for further research to elucidate the mechanisms underlying MNP-induced developmental toxicity.

## 2. Methods

We conducted a comprehensive literature review in PubMed and Web of Science using the following keywords: “microplastics”, “nanoplastics”, “developmental toxicity”, “mammals”, “fetal”, “offspring”, “pregnancy”, “maternal”, and “paternal”. The titles and abstracts of all articles were screened to ensure that their topics were highly relevant to developmental toxicity. Our review included all types of MNPs, both virgin and aged particles, as well as MNPs with surface-charge adsorption. The exclusion criteria were: (1) studies focusing solely on the maternal, placenta, or cell lines without reference to the fetus or offspring; (2) studies involving non-mammalian species; and (3) duplicate articles. The databases were last accessed in November 2024.

## 3. Physicochemical Properties and Exposure Characteristics of MNPs

**Figure 2:**
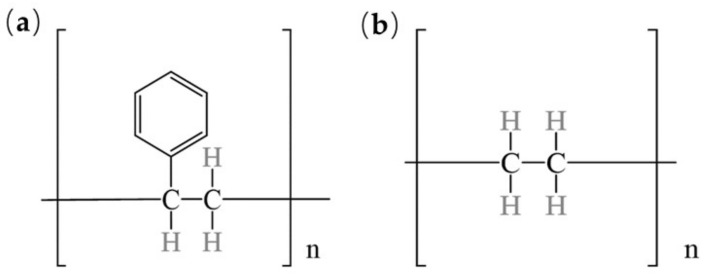
(**a**) Chemical structure of PS; (**b**) chemical structure of PE.

MNPs originate from secondary sources, such as the fragmentation of larger plastic items, and primary sources, including small plastic particles manufactured for consumer and industrial applications [17]. They come in various shapes, including fragments, fibers, foams, pellets, spheres, and films, with fragments and fibers being the most predominant [18]. The common polymer types of MNPs include polyethylene (PE), polypropylene (PP), polyamide (PA), polystyrene (PS), polyvinyl chloride (PVC), polymethyl methacrylate (PMMA) and polyester (PES) [19,20]. As highlighted in the text, polymers such as PE, PP, and PS constitute major components of MNPs [19,20,21], with distinct distribution patterns across ecosystems. In aquatic environments, PE and PP—commonly used in packaging materials and fishing gear—are the dominant MNPs in marine and riverine systems [8,9]. PE’s durability and low density enable it to float on water surfaces, whereas PS fragments into nanoplastics that accumulate in sediments [4,5,6]. Conversely, in terrestrial systems, PES and PA enter soils via sewage sludge application and textile fiber shedding, frequently detected in agricultural fields and urban dust [10]. PVC and PMMA are prevalent in landfills and airborne particulates due to industrial emissions and improper waste disposal [7]. The studies reviewed here primarily focus on PS and PE MNPs (Figure 2).

Humans are exposed to MNPs through various routes, including ingestion of food containing MNPs, inhalation of airborne MNPs, and dermal contact with MNPs found in products, textiles, or dust [7]. Additionally, MNPs were also found in medical injections [22]. Researchers have recently detected various MNPs in human liver, lungs, colon, placenta, stool, sputum, and bloodstream. The accumulation of MNPs in the body can result in a range of adverse health effects, such as hepatotoxicity [23], reproductive toxicity [24], developmental toxicity [16], and neurotoxicity [25].

## 4. Impact of MNPs Exposure on Mammalian Embryo, Fetal, and Placental Development

Exposure to MNPs during sensitive windows of embryonic and fetal development can lead to profound and lasting effects. Studies have demonstrated that MNPs can translocate from maternal systems to the placenta and fetal tissues, including the liver, lung, heart, kidney, and brain [26]. This translocation suggests potential indirect exposure and the possibility of systemic impacts on fetal development. MNPs exposure can lead to a cascade of effects on mammalian embryos, fetuses, and placentas (Table 1). These effects range from direct impacts on developmental progression to indirect effects through the placenta, highlighting the complexity and potential severity of MNPs as developmental toxicant.

### 4.1. Effects on Embryo and Fetal Development

A study by You et al. [27] has shown that exposure to PMMA nanoparticles (NPs) during pregnancy can prolong the developmental period of mouse embryos and significantly reduce the implantation rate. Similarly, Zhang’s and Hu’s research indicated that gestational exposure to PS-NPs increased embryonic resorption rate in mice, pointing to the potential of MNPs to contribute to early fetal intrauterine mortality [28,29]. In addition, a study examining the developmental toxicity of PE microplastics (MPs) also showed reduced live fetal numbers in mouse dams treated with PE-MPs [29]. These findings underscore the profound vulnerability of embryo and fetal survival to MNPs exposure.

In addition to the effects on embryo and fetal survival, MNPs exposure can also cause abnormal fetal weight in experimental mammals. Exposure to varying particle sizes and concentrations of PS-MNPs has been shown to reduce the birth weight of offspring mice, with potential effects persisting postnatally [30,31]. Interestingly, exposure to PS-NPs during gestation resulted in a significant reduction in birth weight for female mice when compared to male offspring [32]. Nonetheless, upon reaching adulthood, both female and male offspring from the PS-NPs-exposed group demonstrated a marked increase in body weight [32]. Additionally, exposure to PE-MPs can influence the sex ratio and reduce body and organ weights in offspring mice [29,33,34]. However, some researchers have indicated that maternal exposure to PE-MNPs does not significantly impact fetal growth [35]. These results indicate that the impact of MNPs exposure during pregnancy on fetal or offspring body weight is influenced by various factors, including the chemical monomers of MNPs, particle size and concentration, as well as the sex of the fetus or offspring. Overall, the impact of PS-MNPs on fetal body weight is greater and may lead to intrauterine growth retardation in mammals.

### 4.2. Effects on Placental Development

The placenta, a temporary organ during pregnancy, is essential for maintaining the health and well-being of both the developing fetus and its mother [36]. However, gestational exposure to MNPs is linked to placental structural abnormalities and functional disorders [26,28], characterized by immune disorder [37], vascular dysfunction [37,38], and abnormal nutrient metabolism and transportation [39], etc.

**Table 1:** Research on the impact of MNPs exposure on mammalian embryo, fetal and placental development.

MNPs			Animals	Exposure			Effects	References
Type	Diameter	Abundance (MNPs/g)		Route	Concentration	Time		
PS	20 nm	2.27 × 10^17^	SD rats	Intratracheal instillation	2.64 × 10^14^ particles	GD 19 to GD 20	Reduced fetal and placental weights; NPs were observed in the placenta, fetal liver, lungs, heart, kidney, and brain	[26]
PS	1 μm	1.82 × 10^12^	C57BL/6J mice	Drink	0.1, 1, 10 mg/L	GD 0.5 to GD 18.5	Increased embryo resorption rate; decreased fetal body and tail length; placental malfunction	[28]
PE	40–48 μm	1.82 × 10^7^–3.14 × 10^7^	ICR mice	Gavage	0.125, 0.5, 2 mg/day	90 days parental exposure and to dams until lactation	Altered numbers of live births per dam, the sex ratio of pups, and body weight of pups	[29]
PS	100 nm	1.82 × 10^15^	Kunming mice	Drink	0.1, 1, 10 mg/L	GD 0 to PND 21	Decreased birth and postnatal body weight in offspring mice	[30]
PS	5 μm, 50 nm	1.46 × 10^10^, 1.46 × 10^16^	CD-1 mice	Drink	10^2^, 10^4^, 10^6^ ng/L	E 0.5 to E 17.5	Fetal growth restriction; reduced umbilical cord length and fetal weights	[31]
PS	80 nm	3.55 × 10^15^	C57BL/6J mice	Oropharyngeal aspiration	1, 5, 25 μg/μL	GD 0 to GD 21	Reduced birth weight of female mice and elevated body weights of adult offspring	[32]
PE	10–45 µm	2.21 × 10^7^–2.01 × 10^9^	ICR mice	Intragastric administration	0.01, 0.1 mg/mouse/day	GD 9 to PND 7	Declining trend in the weight gain and organ weight of neonates; increased serum acetylcholinesterase and glutathione peroxidase levels	[33]
PE	10–150 μm	5.96 × 10^5^–2.01 × 10^9^	Kunming mice	Oral administration	0.4, 4, 40 mg/kg/day	GD 0 to PND 21	Reduced birth and postnatal body weight; reduced number of surviving mice	[34]
PE	740–4990 nm	1.62 × 10^10^–4.96 × 10^12^	CD-1 mice	Drink	10^6^ ng/L	E 0.5 to E 17.5	Increased umbilical artery blood flow	[35]
PS	10 μm	1.82 × 10^9^	C57BL/6-mated BALB/c mice	Intraperitoneal injection	1.25 μg/μL	Injected on GD 5.5 and GD 7.5	Elevated embryo resorption rate; reduced number and diameter of uterine arterioles; reduced percentage of decidual NK cells and increased helper T cells in the placenta; reversed M1/M2 ratio in placental macrophages	[37]
PS	5 μm, 50 nm	1.46 × 10^10^, 1.46 × 10^16^	CD-1 mice	Drink	10^6^ ng/L	GD 0.5 to GD 17.5	Increased umbilical artery blood flow in the MPs-exposed mice and decreased umbilical artery blood flow in the NPs-exposed mice	[38]
PS	5 μm	1.46 × 10^10^	CD-1 mice	Drink	10^2^, 10^4^, 10^6^ ng/L	GD 0.5 to GD 17.5	Reduced fetal weight; decreased lysine and glucose in the placenta; perturbated biotin metabolism, lysine degradation, and glycolysis/gluconeogenesis pathways in placenta	[39]
PS	100 nm	1.82 × 10^15^	C57BL/6 mice	Drink	1, 10 mg/L	GD 0 to GD 17	Reduced fetal weight; abnormal morphologies of cells in the placenta; disturbed cholesterol metabolism and dysregulated complement and coagulation cascades pathways in the placenta	[40]
PS	50 nm	1.46 × 10^16^	C57BL/6 mice	Gavage	25, 50, 100 mg/kg/day	GD 1 to GD 14	Increased miscarriage rates; oxidative stress; decreased mitochondrial membrane potential; and increased apoptosis in trophoblast cells	[41]
PS	30 nm	6.74 × 10^16^	ICR mice	Gavage	0.1, 1, 10 mg/kg/day	GD 0.5 to GD 18.5	Fetal death; reduced weight and the thickness of the trophoblastic layer in the placenta; increased immature red blood cells in the placental vasculature; diminished invasion capabilities of nourishing cells	[42]

GD—gestational day; PND—postnatal day; NK—natural killer.

Hu et al.’s research demonstrated that PS-MPs exposure during pregnancy can alter immune cell populations within the mouse placenta, affecting the balance between pro-inflammatory and anti-inflammatory cytokines [37]. In addition, compared to the control mice, both the quantity and the diameter of uterine arterioles in the placenta were reduced in the exposed mice [37], which indicated vascular dysfunction in the placenta. In particular, Dibbon et al.’s study revealed a 48% increase in umbilical artery blood flow after PS-NPs exposure and a 25% decrease following PS-NPs treatment [38]. Moreover, maternal exposure to PS-NPs through drinking water has also been found to cause significant disturbances in cholesterol metabolism and the complement and coagulation cascade pathways, as revealed by transcriptomic analyses [40]. Similarly, Aghaei et al. [39] found that exposure to PS-MPs can decrease placental levels of essential nutrients like lysine and glucose. These findings suggest that maternal exposure to MNPs during pregnancy may disturb the immune function, angiogenesis, and nutrients metabolism and transportation of the mammalian placenta, potentially impairing fetal growth and development.

The mechanism by which MNPs exposure impairs placental development remains under investigation. Exposure to PS-NPs has been shown to induce oxidative stress and apoptosis in human trophoblast cells, affecting critical signaling pathways and impacting placental and embryonic development [41]. Furthermore, PS-NPs have been linked to reduced expression of mitogen-activated protein kinase 6 (MAP2K6) in placental and trophoblast cells, disrupting placental trophoblast proliferation and migration, and ultimately inhibiting placental and embryonic development [42]. Additional research is still required to elucidate underlying mechanisms.

## 5. Neurodevelopmental Toxicity of MNPs

Due to their small size and high surface area, MNPs can cross biological barriers and accumulate in various tissues, including the developing brain. This section will delve into the studies investigating the impact of MNPs on neurodevelopment, focusing on the critical windows of gestation and early life (Table 2).

**Table 2:** Research on the neurodevelopmental toxicity on mammals.

MNPs			Animals	Exposure			Effects	References
Type	Diameter	Abundance (MNPs/g)		Route	Concentration	Time		
PS	25 nm, 50 nm	1.16 × 10^17^, 1.46 × 10^16^	SD rats	Gavage	0.5, 2.5, 10, 50 mg/ kg	GD 0.5 to GD 17.5, GD 0.5 to GD 21.5	PS-NPs accumulated in brain regions of fetal rats, especially the cerebellum; reduced MBP and MOG expression; decreased myelin thickness; oligodendrocyte apoptosis; impaired motor coordination	[25]
PS	100 nm, 1000 nm	1.82 × 10^15^, 1.82 × 10^12^	C57BL mice	Gavage	1 mg/day	GD 1 to GD 17	Induced anxiety-like behavior; reduced GABA in the prefrontal cortex and amygdala	[43]
PS	5 μm, 50 nm	1.46 × 10^10^, 1.46 × 10^16^	CD-1 mice	Drink	10^6^ ng/L	GD 0.5 to GD 17.5	Decreased middle cerebral artery pulsatility index	[38]
PS	50 nm	1.46 × 10^16^	CD-1 mice	Drink	10^6^ ng/L	GD 0.5 to GD 17.5	Reduced GABA, creatine and glucose in the fetal brain; altered asparagine concentration, with variations influenced by fetal sex	[44]
PS	25, 50 nm	1.16 × 10^17^, 1.46 × 10^16^	SD rats	Gavage	0.5, 2.5, 10, 50 mg/kg	GD 1 to GD 18	Increased levels of IL-1β, IL-6; decreased CAT, SOD and GSH-PX activity; increased MDA content in the prefrontal cortex, hippocampus and striatum	[45]
PS	50, 500 nm	1.46 × 10^16^, 1.46 × 10^13^	C57BL/6J mice	Oral administration	0.5, 10, 100, 500, 1000 μg/day	GD 8 to PND 14	Altered the functioning of NSCs, neural cell compositions, and brain histology; induced neurophysiological and cognitive deficits in a gender-specific manner	[46]
PS	50 nm	1.46 × 10^16^	SD rats	Gavage	2.5 mg/kg/day	GD 0.5 to PND 22	Downregulation of neural developmental proteins and upregulation of inhibitory proteins in the hippocampus; KEGG pathway analysis highlighted ferroptosis enrichment	[47]
PS	50 nm	1.46 × 10^16^	SD rats	Gavage	2.5 mg/kg/day	Gestational, lactational exposure	Diminished cortical thickness; heightened cortical cell proliferation; disrupted neocortical migration; altered monoamine neurotransmitters within the cortex and amino acid neurotransmitters within the hippocampus; widened synaptic clefts; diminished postsynaptic density; deficits in anxiolytic-like behaviors and spatial memory	[48]
PS	100 nm	1.82 × 10^15^	C57BL/6 mice	Drink	10 mg/L	GD 0.5 to PND 21	Defective neural retinal development; delayed retinal vessel development; abnormal ERG responses; oxidative stress in retina	[49]
PS	40, 193 nm	2.84 × 10^16^, 2.53 × 10^14^	C57BL/6J mice	Drink	5, 10 mg/L	Dams: GD 9.5 to PND 28.5, Offspring: PND 28.5 to PND113	Downregulated Gabra2 expression in the brain; abnormal social behavior, anxiety- and depression-like behavior	[50]
PS	2 µm	2.27 × 10^11^	C57BL/6J mice	Drink	1 mg/L	Dams: GD 0.5 to GD 28.5, Offspring: GD 28.5 to PND 168	Reduced dendritic length; impaired social novelty preferences	[51]
PS	200 nm, 1 μm, 5 μm	2.27 × 10^14^, 1.82 × 10^12^, 1.46 × 10^10^	C57BL/6J mice	Oral administration, intragastric injection	5 mg/kg/day	Dams: GD 12.5 to GD 16.5, Offspring: PND 1 to PND 5, PND 14 to PND 18, PND 28 to PND 32	Impair microglia-mediated synaptic pruning; social behavioral defects in adulthood	[52]

GD—gestational day; PND—postnatal day; MBP—myelin basic protein; MOG—myelin oligodendrocyte glycoprotein; GABA—γ-aminobutyric acid; CAT—catalase; SOD—superoxide dismutase; GSH-PX—glutathione peroxidase; MDA—malondialdehyde; NSC—neural stem cell; KEGG—Kyoto Encyclopedia of Genes and Genomes; IL—interleukin; ERG—electroretinogram.

### 5.1. Impact of Gestational Exposure to MNPs on Neurodevelopment

Studies in murine models have shown that maternal ingestion of PS-NPs during pregnancy can lead to transplacental transfer of these particles to the fetal brain, affecting regions such as the cerebellum, hippocampus, thalamus, striatum, and prefrontal cortex [25,43]. This exposure has been associated with alterations in brain metabolism and vascular pulsatility, as well as changes in the expression of proteins critical for myelin formation and neuronal survival [38,44]. The impact of gestational exposure to MNPs extends beyond direct effects on brain structure and function [25]. It also includes the induction of oxidative stress, inflammation, and apoptosis, which can disrupt the delicate balance of neurodevelopment [45]. Furthermore, the exposure has been linked to behavioral changes in offspring, including anxiety-like behavior and impaired social interactions, suggesting that MNPs may have long-lasting effects on brain function and behavior [43].

### 5.2. Impact of Exposure to MNPs During Both Pregnancy and Lactation Period on Neurodevelopment

PS-NPs administered via gavage during the gestation and lactation period were found to disrupt the structural integrity and neuronal composition of the developing brain, leading to functional impairments in hippocampal neural stem cells (NSCs) [46]. The study further demonstrated that aberrant brain development leads to neurophysiological and cognitive deficits, with these impairments manifesting in a sex-dependent manner [46]. Studies have also found that maternal exposure to PS-NPs can reduce the expression of neurodevelopmental proteins and an increase in inhibitory proteins in the hippocampus, which is crucial for learning and memory [47]. Moreover, simultaneous exposure during the prenatal and postnatal periods has been found to result in significant changes in neurotransmitter levels, affecting both monoamine and amino acid neurotransmitter systems [48]. These changes have been correlated with impaired cortical development and disrupted neuronal migration. Maternal exposure to PS-NPs has also been linked to impaired neural retinal development in neonatal mice, with particle deposition in retinal tissue leading to reduced retinal ganglion and bipolar cells, hindered retinal vascular development, and increased oxidative stress [49].

### 5.3. Long-Term Impact of MNPs Exposure on Neurodevelopment

Shin [50] and So [51] observed that parental and progeny exposure to PS-MNPs significantly inhibited neuronal growth, modulated cellular proliferation, and enhanced apoptosis. Mice chronically exposed to MNPs from fetal to adult life displayed signs of anxiety- and depression-like behaviors, and atypical social interactions [50,51]. And downregulation of genes associated with brain development, such as γ-aminobutyric acid type A receptor subunit α2 (GABRA2), was found in their brain since the fetal stage [50].

Interestingly, unlike other maternal exposures, neonatal exposure to PS-NPs has been shown to disrupt microglial autophagy and energy metabolism, which in turn impairs the microglia-mediated process of synaptic pruning [52]. This early disruption can have lasting effects, leading to social behavioral abnormalities in adulthood, underscoring the potential for MNPs to exert profound and enduring impacts on brain development and subsequent behavioral outcomes.

## 6. The Impact of Parental Exposure to MNPs on Other Tissues in Offspring

### 6.1. Impact on the Liver in Offspring

Parental exposure to MNPs has been linked to significant liver and metabolic perturbations in mammalian offspring (Table 3) [30,53,54,55,56]. High-dose PS-NPs exposure in Kunming mice during pregnancy and lactation has led to decreased liver weight and hepatic metabolism disruptions in male offspring [30], while gestational exposure to PS-NPs induced liver steatosis in female offspring, alongside the upregulation of key metabolic genes in the liver [53]. Maternal PS-MPs exposure has also been shown to predispose offspring to metabolic disorders, with hepatic lipid accumulation observed in adult females and intergenerational metabolic impacts extending to subsequent generations [54,55]. Furthermore, extended paternal exposure to PS-MPs has resulted in lipid class alterations and dysregulation of lipid metabolism in the liver and plasma of offspring, with specific increases in certain phospholipids associated with non-alcoholic fatty liver disease [56].

### 6.2. Impact on Intestinal Health in Offspring

Parental exposure to MNPs is related to intestinal health perturbations in offspring (Table 3) [32,54,57,58]. Sun et al. [57] reported that paternal exposure to PE-NPs led to microbiota imbalances, with specific taxa changes correlating to spermatogenic dysfunction. However, no such alterations were observed in ICR mice offspring following maternal PS-MPs exposure [54]. At the same time, in the study by Zhang et al. [58], they found the alterations in the intestinal microbiota composition of offspring from C57BL/6 J mice parents exposed to PS-NPs contributed to the observed changes in body weight. In a subsequent investigation, histological alterations within the small intestine were observed, alongside an increase in reactive oxygen species (ROS) and a concomitant reduction in the levels of glutathione peroxidase 4 (GPx4), ferritin heavy chain 1 (FTH1), and ferritin light chain (FTL) proteins [32]. These studies collectively indicate that MNPs can disrupt the intestinal microbiome and health, potentially triggering cellular damage processes such as ferroptosis.

**Table 3:** Research on the impact of parental exposure to MNPs on offspring’s other tissues.

MNPs			Animals	Exposure			Effects	References
Type	Diameter	Abundance (MNPs/g)		Route	Concentration	Time		
***Liver and metabolic functions***								
PS	100 nm	1.82 × 10^15^	Kunming mice	Drink	0.1, 1 and 10 mg/L	GD 0 to PND 21	Reduced liver weight in male offspring; oxidative stress, inflammatory cell infiltration, and disturbed glycometabolism in the liver of male offspring	[30]
PS	71.09 ± 6.63 nm	5.30 × 10^15^	C57BL/6J mice	Oropharyngeal aspiration	1, 5, 25 μg/μL	GD 0 to PND 0	Hepatic steatosis in adult female offspring	[53]
PS	5 μm	1.46 × 10^10^	ICR mice	Drink	100, 1000 μg/L	Gestational, lactational exposure for 6 weeks	Hepatic transcriptome and serum metabolite changes in F1 generation; potential of hepatic lipid accumulation in adult F1 generation	[54]
PS	0.5, 5 μm	1.46 × 10^13^– 1.46 × 10^10^	ICR mice	Drink	100, 1000 μg/L	GD 0 to PND 0	Altered serum TG, TC, HDL-C and LDL-C levels and hepatic TC, TG levels in F1 generation; FA metabolism disorder in the F1 generation	[55]
PS	40–100 μm	1.82 × 10^6^, 2.84 × 10^7^	C57BL/6J mice	Feed	50, 500 mg/kg MP-contained food	Paternal exposure for 21 weeks before pregnancy	Dysregulated lipid metabolism in the liver and plasma of F1 generationin in a dose-, gender-, and tissue-specific pattern	[56]
***Intestinal health***								
PE	200 nm	2.52 × 10^14^	C57BL/6J mice	Gavage	2 mg/L	Paternal exposure for 35 days before pregnancy	Gut microbial dysbiosis in F0 and F1 generation	[57]
PS	5 μm	1.46 × 10^10^	ICR mice	Drink	100, 1000 μg/L	Gestational, lactational exposure for 6 weeks	Gut microbiota dysbiosis and gut barrier dysfunction in dams	[54]
PS, PP	50 nm (PS), 500 nm (PP)	1.46 × 10^16^, 1.68 × 10^13^	C57BL/6J mice	Feed	0.5, 10, 100, 500 μg/day	GD 8 to PND 14	Induced shifts in the distribution of intestinal microbes.	[58]
PS	80 nm	3.55 × 10^15^	C57/BL6J mice	Oropharyngeal aspiration	1, 5, 25 μg/μL	GD 0 to GD 21	Altered small intestine morphology; induced oxidative stress and initiated ferroptosis in the small intestines; female offspring showed higher small intestinal damage than males	[32]
***Reproductive health***								
PS	100 nm	1.82 × 10^15^	Kunming mice	Drink	0.1, 1, 10 mg/L	GD 0 to PND 21	Diminished testis weight; decreased sperm counts; disrupted seminiferous epithelium; oxidative stress in the testis	[30]
PE	200 nm	2.52 × 10^14^	C57BL/6J mice	Gavage	2 mg/L	Paternal exposure for 35 days before pregnancy	Abnormal growth phenotypes and sex hormone levels; histological damage in the testicular tissue; reduced total sperm counts and motility; sperm abnormality; altered microRNA profiles in the sperm	[57]
PS	0.5 μm	1.46 × 10^13^	ICR mice	Drink	0.5, 5, 50 mg/L	GD 1 to PND 35 or PND 70	Testis development disorder; spermatogenesis dysfunction	[59]
PS	1 μm	1.82 × 10^12^	ICR mice	Gavage	1.357 ng/g/day, 1.357 μg/g/day	F0 PND 0 to PND 21	Decreased sperm count and viability in F1 male offspring; decreased sperm count in the F2 male offspring	[60]
PE	10–150 μm	5.96 × 10^5^– 2.01 × 10^9^	Kunming mice	Oral administration	0.4, 4, 40 mg/kg/day	GD 0 to PND 21	Reduced oocyte maturation, fertilization rate, and embryonic development in female offspring; oxidative stress in the ovaries	[34]
***Spleen immunity***								
PS	1 μm	1.82 × 10^12^	ICR mice	Intragastric administration	1.36 × 10^−6^, 1.36 × 10^−3^ mg/g/day	GD 0 to PND 21	Increased spleen weight in offspring; elevated number of B cells, Th cells and Tregs in spleen of male offspring; elevated ratio of Th17/Tregs and Th1/Th2 cells in F1 male offspring	[61]
PE	16.9 ± 1.9 μm	4.17 × 10^8^	ICR mice	Gavage	0.125, 0.5, 2 mg/day	90 days parental exposure and to dams until lactation	Decreased proportion of Tregs within the spleen in the female pups; increased proportion of Th cells within the spleen in both sexes of pups; inhibited maturation of dendritic cells in splenocytes of male pups, while it was enhanced in female pups	[29]
***Bone and skeletal muscle health***								
PS	100 nm	1.82 × 10^15^	C57BL/6 mice	Drink	1, 10 mg/L	GD 0 to GD 17	Disturbed cholesterol metabolism, complement and coagulation cascade and muscle tissue formation in fetal skeletal muscle	[40]
PS	1 μm	1.82 × 10^12^	SD rats	Gavage	2 mg/kg/day	28 days	Shortened tibial length and altered blood calcium and phosphorus metabolism; altered expression of the transcription factors involved in chondrocyte proliferation, differentiation, apoptosis, and matrix secretion in tibial proximal growth plate tissue	[62]
[63]								
PS	20 nm	2.27 × 10^17^	SD rats	Intratracheal instillation	2.64 × 10^14^ particles	GD 19 to GD 20	Increased heart weight and vascular dysfunction in the aorta of dams; vascular dysfunction in the radial artery of the uterus in offspring; dysfunction of the fetal heart, fetal aorta, and umbilical artery	

GD—gestational day; PND—postnatal day; TG—triacylglycerol; TC—cholesterol; HDL-C—high-density lipoprotein cholesterol; LDL-C—low-density lipoprotein cholesterol; FA—fatty acid; Th cells—T helper cells; Tregs—regulatory T cells; Th17 cells—T helper cells 17; Th1 cells—T helper cells 1; Th2 cells—T helper cells 2.

### 6.3. Impact on Reproductive Health in Offspring

Studies reveal that maternal or paternal exposure to MNPs can lead to spermatogenesis disruption, hormonal imbalances, and testicular damage in male mice offspring, potentially mediated by oxidative stress and immune responses (Table 3) [30,57,59]. Additionally, there is evidence of transgenerational impacts, including impaired oocyte maturation, fertilization rate and embryonic development in female mice offspring, suggesting a broad range of reproductive dysfunctions associated with MNPs exposure [60].

### 6.4. Disruption of Spleen Immunity in Offspring

Studies indicate that parental exposure to PS or PE MNPs can alter spleen immunity in mice offspring, mainly manifesting as abnormal changes in the proportion of different populations of lymphocytes in the spleen, such as B cells and regulatory T cells (Tregs) [29,61]. Research has revealed that alterations in immune status are transmissible to the F1 generation but do not persist beyond it, suggesting the absence of transgenerational inheritance [61].

### 6.5. Impact of Parental MNPs Exposure on Bone and Skeletal Muscle Health in Offspring

It has been shown that maternal exposure to PS-NPs through drinking water modulated the expression of genes associated with muscle tissue development and lipid metabolism in the fetal skeletal muscle [40]. In addition, PS-MPs could induce growth retardation and longitudinal bone damage in early development [62]. These indicate that MNPs could adversely affect the early development of bone and skeletal growth in mice.

### 6.6. Impact on Cardiovascular Function in Offspring

One study showed that maternal exposure to PS-NPs resulted in cardiovascular dysfunction in both the mother and her offspring, affecting the heart and specific arteries [63].

## 7. Conclusions and Perspective

**Figure 3:**
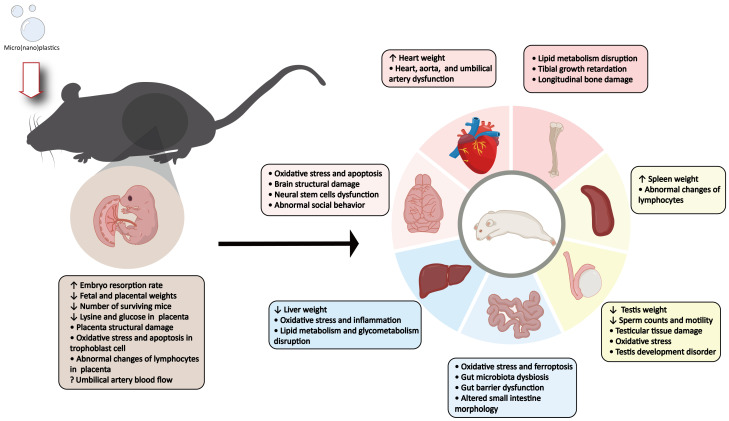
The adverse effects of maternal exposure to micro(nano)plastics on the fetus and placenta, as well as the adverse effects on various organs and physiological processes in offspring mice.

This review comprehensively examines the developmental toxicity of MNPs in mammals, uncovering concerning findings regarding their effects on mammalian offspring (Figure 3). The evidence indicates that MNPs can translocate from maternal circulation to the fetus, disrupt embryonic and placental development, and lead to neurodevelopmental deficits and tissue dysfunction in offspring. These findings are corroborated by human observational data, which show inverse correlations between placental MNP levels and neonatal anthropometric parameters (e.g., birth weight and head circumference) and associations between amniotic fluid MNP concentrations and shortened gestational age [64,65]. Overall, these results underscore the pressing concern posed by MNPs to mammalian development.

Despite these concerning findings, significant limitations exist in the reviewed studies. Firstly, most studies rely on animal models, which may not fully translate to human health due to species-specific differences in placental structure and function, as well as variations in metabolic processes. Secondly, experimental designs often involve high concentrations and short-term exposures, which may overestimate the developmental toxic effects compared to more realistic, low-dose, long-term exposure scenarios in humans. Thirdly, the bioavailability and long-term accumulation of MNPs in tissues remain poorly understood, making it challenging to accurately assess their potential health impacts on the development of mammals. Additionally, existing studies lack sufficient investigation into the interactions between MNPs and other environmental contaminants. These interactions are crucial as MNPs can adsorb and transport harmful substances, including heavy metals, persistent organic pollutants, and pathogens. Understanding these combined toxic effects is crucial for a comprehensive assessment of MNP-related developmental toxicity risks. Future research should focus on elucidating the long-term developmental toxic effects and mechanisms of low-dose MNP exposure, investigating the bioavailability and accumulation of MNPs in tissues, and exploring the combined developmental toxic effects of MNPs with other environmental contaminants. Moreover, the potential for MNPs to induce transgenerational impacts, as suggested by initial evidence of epigenetic modifications, warrants thorough investigation. Finally, there is an urgent need to strengthen epidemiological studies on the developmental toxicity of MNPs in human populations.

Given these considerations, immediate action is essential. Reducing plastic use during pregnancy and infancy, particularly plastic tableware and cups, is a targeted measure to mitigate the developmental toxicity of MNPs. Continued international cooperation and evidence-based policy development are vital for addressing plastic pollution and safeguarding human health. Specific strategies include reducing single-use plastics, promoting biodegradable alternatives, and strengthening international collaboration for standardized regulation of plastic production and recycling, as well as MNPs monitoring. These actions, combined with robust epidemiological surveys and evidence-based policy frameworks, provide actionable guidance for mitigating the developmental toxicity of MNPs while fostering continued research and global cooperation.

## Data Availability

No new data were created or analyzed in this study. Data sharing is not applicable to this article.

## References

[1] Petersen F., Hubbart J.A. (2021). The Occurrence and Transport of Microplastics: The State of the Science. Sci. Total Environ..

[2] Lim X. (2021). Microplastics Are Everywhere—But Are They Harmful?. Nature.

[3] Geyer R., Jambeck J.R., Law K.L. (2017). Production, Use, and Fate of All Plastics Ever Made. Sci. Adv..

[4] Andrady A.L. (2011). Microplastics in the Marine Environment. Mar. Pollut. Bull..

[5] Cole M., Lindeque P., Halsband C., Galloway T.S. (2011). Microplastics as Contaminants in the Marine Environment: A Review. Mar. Pollut. Bull..

[6] Gigault J., Halle A.T., Baudrimont M., Pascal P.-Y., Gauffre F., Phi T.-L., El Hadri H., Grassl B., Reynaud S. (2018). Current Opinion: What Is a Nanoplastic?. Environ. Pollut..

[7] Zhu L., Kang Y., Ma M., Wu Z., Zhang L., Hu R., Xu Q., Zhu J., Gu X., An L. (2024). Tissue Accumulation of Microplastics and Potential Health Risks in Human. Sci. Total Environ..

[8] Jiang B., Kauffman A.E., Li L., McFee W., Cai B., Weinstein J., Lead J.R., Chatterjee S., Scott G.I., Xiao S. (2020). Health Impacts of Environmental Contamination of Micro- and Nanoplastics: A Review. Environ. Health Prev. Med..

[9] Wang Z., Kang S., Zhang Y., Luo X., Kang Q., Chen P., Guo J., Hu Z., Yang Z., Zheng H. (2024). Microplastics in Glaciers of Tibetan Plateau: Characteristics and Potential Sources. Sci. Total Environ..

[10] Vdovchenko A., Resmini M. (2024). Mapping Microplastics in Humans: Analysis of Polymer Types, and Shapes in Food and Drinking Water-A Systematic Review. Int. J. Mol. Sci..

[11] Zantis L.J., Carroll E.L., Nelms S.E., Bosker T. (2021). Marine Mammals and Microplastics: A Systematic Review and Call for Standardisation. Environ. Pollut..

[12] Liu M., Liu J., Xiong F., Xu K., Pu Y., Huang J., Zhang J., Pu Y., Sun R., Cheng K. (2023). Research Advances of Microplastics and Potential Health Risks of Microplastics on Terrestrial Higher Mammals: A Bibliometric Analysis and Literature Review. Environ. Geochem. Health.

[13] Ayala F., Zeta-Flores M., Ramos-Baldárrago S., Tume-Ruiz J., Rangel-Vega A., Reyes E., Quinde E., De-la-Torre G.E., Lajo-Salazar L., Cárdenas-Alayza S. (2023). Terrestrial Mammals of the Americas and Their Interactions with Plastic Waste. Environ. Sci. Pollut. Res. Int..

[14] Gallitelli L., Battisti C., Pietrelli L., Scalici M. (2022). Anthropogenic Particles in Coypu (Myocastor Coypus; Mammalia, Rodentia)’ Faeces: First Evidence and Considerations about Their Use as Track for Detecting Microplastic Pollution. Environ. Sci. Pollut. Res. Int..

[15] Tyl R.W., Wexler P. (2014). Toxicity Testing, Developmental. Encyclopedia of Toxicology.

[16] Huang W., Mo J., Li J., Wu K. (2024). Exploring Developmental Toxicity of Microplastics and Nanoplastics (MNPS): Insights from Investigations Using Zebrafish Embryos. Sci. Total Environ..

[17] Sulaiman R.N.R., Bakar A.A., Ngadi N., Kahar I.N.S., Nordin A.H., Ikram M., Nabgan W. (2023). Microplastics in Malaysia’s Aquatic Environment: Current Overview and Future Perspectives. Glob. Chall..

[18] Kiran B.R., Kopperi H., Venkata Mohan S. (2022). Micro/Nano-Plastics Occurrence, Identification, Risk Analysis and Mitigation: Challenges and Perspectives. Rev. Environ. Sci. Biotechnol..

[19] Fan P., Tan W., Yu H. (2022). Effects of Different Concentrations and Types of Microplastics on Bacteria and Fungi in Alkaline Soil. Ecotoxicol. Environ. Saf..

[20] Almeida J.M., Singdahl-Larsen C., Buenaventura N., Gomes L., Morgado V., Olsen M., Bettencourt da Silva R., Palma C. (2023). Assessment and Comparison of Microplastic Contamination in Atlantic Navigation Routes with Known Uncertainty. Environ. Sci. Technol..

[21] Dybka-Stępień K., Antolak H., Kmiotek M., Piechota D., Koziróg A. (2021). Disposable Food Packaging and Serving Materials—Trends and Biodegradability. Polymers.

[22] Liu Z., Wang D., Liu Z., Xu C., Zhang Y., Liu P. (2024). Microplastic Injection? Identification and Quantification of Plastic Particles in Medical Injections. Sci. Total Environ..

[23] Ge Y., Yang S., Zhang T., Wan X., Zhu Y., Yang F., Yin L., Pu Y., Liang G. (2023). The Hepatotoxicity Assessment of Micro/Nanoplastics: A Preliminary Study to Apply the Adverse Outcome Pathways. Sci. Total Environ..

[24] Yang S., Li M., Kong R.Y.C., Li L., Li R., Chen J., Lai K.P. (2023). Reproductive Toxicity of Micro- and Nanoplastics. Environ. Int..

[25] Zhang Y., Tian L., Chen J., Liu X., Li K., Liu H., Lai W., Shi Y., Lin B., Xi Z. (2024). Selective Bioaccumulation of Polystyrene Nanoplastics in Fetal Rat Brain and Damage to Myelin Development. Ecotoxicol. Environ. Saf..

[26] Fournier S.B., D’Errico J.N., Adler D.S., Kollontzi S., Goedken M.J., Fabris L., Yurkow E.J., Stapleton P.A. (2020). Nanopolystyrene Translocation and Fetal Deposition after Acute Lung Exposure during Late-Stage Pregnancy. Part. Fibre Toxicol..

[27] You H.-J., Jo Y.-J., Kim G., Kwon J., Yoon S.-B., Youn C., Kim Y., Kang M.-J., Cho W.-S., Kim J.-S. (2024). Disruption of Early Embryonic Development in Mice by Polymethylmethacrylate Nanoplastics in an Oxidative Stress Mechanism. Chemosphere.

[28] Zhang R., Feng Y., Nie P., Wang W., Wu H., Wan X., Xu H., Fu F. (2024). Polystyrene Microplastics Disturb Maternal Glucose Homeostasis and Induce Adverse Pregnancy Outcomes. Ecotoxicol. Environ. Saf..

[29] Park E.-J., Han J.-S., Park E.-J., Seong E., Lee G.-H., Kim D.-W., Son H.-Y., Han H.-Y., Lee B.-S. (2020). Repeated-Oral Dose Toxicity of Polyethylene Microplastics and the Possible Implications on Reproduction and Development of the next Generation. Toxicol. Lett..

[30] Huang T., Zhang W., Lin T., Liu S., Sun Z., Liu F., Yuan Y., Xiang X., Kuang H., Yang B. (2022). Maternal Exposure to Polystyrene Nanoplastics during Gestation and Lactation Induces Hepatic and Testicular Toxicity in Male Mouse Offspring. Food Chem. Toxicol..

[31] Aghaei Z., Sled J.G., Kingdom J.C., Baschat A.A., Helm P.A., Jobst K.J., Cahill L.S. (2022). Maternal Exposure to Polystyrene Micro- and Nanoplastics Causes Fetal Growth Restriction in Mice. Environ. Sci. Technol. Lett..

[32] Tang J., Bu W., Hu W., Zhao Z., Liu L., Luo C., Wang R., Fan S., Yu S., Wu Q. (2023). Ferroptosis Is Involved in Sex-Specific Small Intestinal Toxicity in the Offspring of Adult Mice Exposed to Polystyrene Nanoplastics during Pregnancy. ACS Nano.

[33] Song Y., Kim C. (2021). Toxicities Demonstrated in Dams and Neonates Following Intragastric Intubation of Polyethylene Microplastics to Pregnant Mice. J. Environ. Health Sci..

[34] Zhang Y., Wang X., Zhao Y., Zhao J., Yu T., Yao Y., Zhao R., Yu R., Liu J., Su J. (2023). Reproductive Toxicity of Microplastics in Female Mice and Their Offspring from Induction of Oxidative Stress. Environ. Pollut..

[35] Hanrahan J., Steeves K.L., Locke D.P., O’Brien T.M., Maekawa A.S., Amiri R., Macgowan C.K., Baschat A.A., Kingdom J.C., Simpson A.J. (2024). Maternal Exposure to Polyethylene Micro- and Nanoplastics Impairs Umbilical Blood Flow but Not Fetal Growth in Pregnant Mice. Sci. Rep..

[36] Cindrova-Davies T., Sferruzzi-Perri A.N. (2022). Human Placental Development and Function. Semin. Cell Dev. Biol..

[37] Hu J., Qin X., Zhang J., Zhu Y., Zeng W., Lin Y., Liu X. (2021). Polystyrene Microplastics Disturb Maternal-Fetal Immune Balance and Cause Reproductive Toxicity in Pregnant Mice. Reprod. Toxicol..

[38] Dibbon K.C., Mercer G.V., Maekawa A.S., Hanrahan J., Steeves K.L., Ringer L.C.M., Simpson A.J., Simpson M.J., Baschat A.A., Kingdom J.C. (2024). Polystyrene Micro- and Nanoplastics Cause Placental Dysfunction in Mice†. Biol. Reprod..

[39] Aghaei Z., Mercer G.V., Schneider C.M., Sled J.G., Macgowan C.K., Baschat A.A., Kingdom J.C., Helm P.A., Simpson A.J., Simpson M.J. (2022). Maternal Exposure to Polystyrene Microplastics Alters Placental Metabolism in Mice. Metabolomics.

[40] Chen G., Xiong S., Jing Q., van Gestel C.A.M., van Straalen N.M., Roelofs D., Sun L., Qiu H. (2023). Maternal Exposure to Polystyrene Nanoparticles Retarded Fetal Growth and Triggered Metabolic Disorders of Placenta and Fetus in Mice. Sci. Total Environ..

[41] Wan S., Wang X., Chen W., Wang M., Zhao J., Xu Z., Wang R., Mi C., Zheng Z., Zhang H. (2024). Exposure to High Dose of Polystyrene Nanoplastics Causes Trophoblast Cell Apoptosis and Induces Miscarriage. Part. Fibre Toxicol..

[42] Lv J., He Q., Yan Z., Xie Y., Wu Y., Li A., Zhang Y., Li J., Huang Z. (2024). Inhibitory Impact of Prenatal Exposure to Nano-Polystyrene Particles on the MAP2K6/P38 MAPK Axis Inducing Embryonic Developmental Abnormalities in Mice. Toxics.

[43] Yang D., Zhu J., Zhou X., Pan D., Nan S., Yin R., Lei Q., Ma N., Zhu H., Chen J. (2022). Polystyrene Micro- and Nano-Particle Coexposure Injures Fetal Thalamus by Inducing ROS-Mediated Cell Apoptosis. Environ. Int..

[44] Mercer G.V., Harvey N.E., Steeves K.L., Schneider C.M., Sled J.G., Macgowan C.K., Baschat A.A., Kingdom J.C., Simpson A.J., Simpson M.J. (2023). Maternal Exposure to Polystyrene Nanoplastics Alters Fetal Brain Metabolism in Mice. Metabolomics.

[45] Zhang Y.-P., Tian L., Xie X.-Q., Wang Y.-T., Lyu P., Xi Z.-G. (2022). [Effects of nanopolystyrene nanoplastic exposure on the development and neurotoxicity of fetal rats during gestation]. Zhongguo Ying Yong Sheng Li Xue Za Zhi.

[46] Jeong B., Baek J.Y., Koo J., Park S., Ryu Y.-K., Kim K.-S., Zhang S., Chung C., Dogan R., Choi H.-S. (2022). Maternal Exposure to Polystyrene Nanoplastics Causes Brain Abnormalities in Progeny. J. Hazard. Mater..

[47] Chen J., Zhang Y., Liu X., Li K., Liu H., Lai W., Shi Y., Xi Z., Yan L., Tian L. (2024). Effects of Exposure to Nano-Plastic Drinking during Pregnancy on Cognitive Related Proteins in Offspring of SD Rats. Environ. Pollut. Bioavailab..

[48] Tian L., Zhang Y., Chen J., Liu X., Nie H., Li K., Liu H., Lai W., Shi Y., Xi Z. (2024). Effects of Nanoplastic Exposure during Pregnancy and Lactation on Neurodevelopment of Rat Offspring. J. Hazard. Mater..

[49] Xiong S., He J., Qiu H., van Gestel C.A.M., He E., Qiao Z., Cao L., Li J., Chen G. (2024). Maternal Exposure to Polystyrene Nanoplastics Causes Defective Retinal Development and Function in Progeny Mice by Disturbing Metabolic Profiles. Chemosphere.

[50] Shin H.S., Lee S.H., Moon H.J., So Y.H., Lee H.R., Lee E.-H., Jung E.-M. (2023). Exposure to Polystyrene Particles Causes Anxiety-, Depression-like Behavior and Abnormal Social Behavior in Mice. J. Hazard. Mater..

[51] So Y.H., Shin H.S., Lee S.H., Moon H.J., Jang H.J., Lee E.-H., Jung E.-M. (2023). Maternal Exposure to Polystyrene Microplastics Impairs Social Behavior in Mouse Offspring with a Potential Neurotoxicity. Neurotoxicology.

[52] Zou L., Xu X., Wang Y., Lin F., Zhang C., Liu R., Hou X., Wang J., Jiang X., Zhang Q. (2024). Neonatal Exposure to Polystyrene Nanoplastics Impairs Microglia-Mediated Synaptic Pruning and Causes Social Behavioral Defects in Adulthood. Environ. Sci. Technol..

[53] Wang X., Zhao Z., Wang X., Hu W., Luo C., Chu X., Qian M., Wang R., Yu S., Wu Q. (2023). Effects of Polystyrene Nanoplastic Gestational Exposure on Mice. Chemosphere.

[54] Luo T., Wang C., Pan Z., Jin C., Fu Z., Jin Y. (2019). Maternal Polystyrene Microplastic Exposure during Gestation and Lactation Altered Metabolic Homeostasis in the Dams and Their F1 and F2 Offspring. Environ. Sci. Technol..

[55] Luo T., Zhang Y., Wang C., Wang X., Zhou J., Shen M., Zhao Y., Fu Z., Jin Y. (2019). Maternal Exposure to Different Sizes of Polystyrene Microplastics during Gestation Causes Metabolic Disorders in Their Offspring. Environ. Pollut..

[56] Deng Y., Chen H., Huang Y., Wang Q., Chen W., Chen D. (2022). Polystyrene Microplastics Affect the Reproductive Performance of Male Mice and Lipid Homeostasis in Their Offspring. Environ. Sci. Technol. Lett..

[57] Sun J., Teng M., Zhu W., Zhao X., Zhao L., Li Y., Zhang Z., Liu Y., Bi S., Wu F. (2024). MicroRNA and Gut Microbiota Alter Intergenerational Effects of Paternal Exposure to Polyethylene Nanoplastics. ACS Nano.

[58] Jeong B., Kim J.-S., Kwon A.R., Lee J., Park S., Koo J., Lee W.S., Baek J.Y., Shin W.-H., Lee J.-S. (2024). Maternal Nanoplastic Ingestion Induces an Increase in Offspring Body Weight through Altered Lipid Species and Microbiota. Environ. Int..

[59] Zhao T., Shen L., Ye X., Bai G., Liao C., Chen Z., Peng T., Li X., Kang X., An G. (2023). Prenatal and Postnatal Exposure to Polystyrene Microplastics Induces Testis Developmental Disorder and Affects Male Fertility in Mice. J. Hazard. Mater..

[60] Dou Y., Zhang M., Zhang H., Zhang C., Feng L., Hu J., Gao Y., Yuan X.-Z., Zhao Y., Zhao H. (2024). Lactating Exposure to Microplastics at the Dose of Infants Ingested during Artificial Feeding Induced Reproductive Toxicity in Female Mice and Their Offspring. Sci. Total Environ..

[61] Shang Q., Wu H., Wang K., Zhang M., Dou Y., Jiang X., Zhao Y., Zhao H., Chen Z.-J., Wang J. (2024). Exposure to Polystyrene Microplastics during Lactational Period Alters Immune Status in Both Male Mice and Their Offspring. Sci. Total Environ..

[62] Zhang Q., Lang Y., Tang X., Cheng W., Cheng Z., Rizwan M., Xie L., Liu Y., Xu H., Liu Y. (2024). Polystyrene Microplastic-Induced Endoplasmic Reticulum Stress Contributes to Growth Plate Endochondral Ossification Disorder in Young Rat. Environ. Toxicol..

[63] Cary C.M., Fournier S.B., Adams S., Wang X., Yurkow E.J., Stapleton P.A. (2024). Single Pulmonary Nanopolystyrene Exposure in Late-Stage Pregnancy Dysregulates Maternal and Fetal Cardiovascular Function. Toxicol. Sci..

[64] Amereh F., Amjadi N., Mohseni-Bandpei A., Isazadeh S., Mehrabi Y., Eslami A., Naeiji Z., Rafiee M. (2022). Placental Plastics in Young Women from General Population Correlate with Reduced Foetal Growth in IUGR Pregnancies. Environ. Pollut..

[65] Xue J., Xu Z., Hu X., Lu Y., Zhao Y., Zhang H. (2024). Microplastics in Maternal Amniotic Fluid and Their Associations with Gestational Age. Sci. Total Environ..

